# Application of Superabsorbent Spacer Fabrics as Exuding Wound Dressing

**DOI:** 10.3390/polym10020210

**Published:** 2018-02-22

**Authors:** Yadie Yang, Hong Hu

**Affiliations:** Institute of Textiles and Clothing, The Hong Kong Polytechnic University, Hung Hom, Kowloon, Hong Kong, China; tiffany.yang@connect.polyu.hk

**Keywords:** wound dressing, exuding wound, superabsorbent fabric, spacer fabric

## Abstract

Exuding wound care requires a dressing to quickly absorb exudates and properly manage moisture during the healing process. In this study, the superabsorbent spacer fabrics were designed and fabricated for application in exuding wound dressings. The fabric structure consists of three layers, including two outer hydrophobic layers made of polyester/spandex yarns and one superabsorbent middle layer made of superabsorbent yarns. In order to confirm the performance of these superabsorbent spacer fabrics, their dressing properties were tested and compared with two commercial foam dressings. The results showed that all the superabsorbent spacer fabrics had much faster wetting speeds (less than 2 s) than the foam dressings (6.04 s for Foam A and 63.69 s for Foam B). The absorbency of the superabsorbent spacer fabrics was at least twice higher than that of the foam dressings. The air permeability of the superabsorbent spacer fabrics (higher than 15 mL/s/cm^2^ at 100 Pa) was much higher than that of the foam dressings which had a too low permeability to be measured by the testing device. In addition, the water vapor permeability, thermal insulation, and conformability of superabsorbent spacer fabrics were comparable to foam dressings. The study indicates that the superabsorbent spacer fabrics are suitable for exuding wound dressing applications.

## 1. Introduction

Wound management and dressing selection are important aspects of healthcare. Historically, it was believed that a dry wound environment could promote healing and inhibit infection. In 1962, Winter [1] found that if the formation of scab was prevented, the rate of epithelization could be markedly increased. This finding has led to the formation of a new concept to treat wounds, that is, a moist environment facilitates wound healing. Since then, occlusive dressings have been widely used based on this moist-healing concept.

No single dressing could adapt to all kinds of wounds, so wound dressing should be selected according to the wound type. Wounds can be classified into four categories based on their appearance and stage of healing: necrotic, sloughy, granulating, and epithelializing [2]. This study focuses on the application of superabsorbent spacer fabrics as dressings for granulating wounds, which often produce substantial exudates and induce severe heat loss especially when wound area is large. At this stage, a warm, moist environment and minimal disturbance are required to promote wound healing [3]. 

An ideal dressing for exuding wounds requires quick and large absorbency, good thermal insulating property, moisture management and mechanical property. A fast wetting property helps dressing to absorb exudate immediately and avoid maceration of surrounding skin. In addition, a high absorbency is desired for absorbing all liquid emitted from wound. With high absorbency, the change frequency of wound dressing could be considerably reduced. Thus, the possibility to disturb the wound would be lowered. A high heat keeping rate is also required for maintaining the wound at a normal body temperature, allowing an optimal cellular function. At low temperature, vasoconstriction may cause oxygen delivery reduction, which can slow down growth factor production and mitotic activity [3]. Another important factor is the mechanical property of the dressing. When applying to a wound, the dressing should adapt to the body shape of the wound site. The wound dressing should be elastic and conformable, especially when applied on a joint site. The moist-healing concept requires wound tissues to be physiologically moist, neither dry nor covered in fluid [4]. It is crucial that the moisture vaporization should be maintained in a reasonable range to keep the wound surface moist and avoid maceration of intact skin. The maceration of skin may cause skin breakdown, raised infection potential, and healing delay [5]. In addition, an oxygen-permeable dressing could accelerate wound healing by promoting fibroblast proliferation and collagen synthesis [6,7,8]. 

The current commercial dressings have some limitations. Cotton gauze is the most used wound dressing, but its high evaporation and low absorbency limit its application [9,10,11]. Hydrofiber dressing and alginate dressing are normally non-woven materials, and they form gels when contacted with wound fluid [12,13]. Although hydrofiber dressing and alginate dressing have high absorbency, they are non-occlusive and need a secondary dressing on their surface to keep a moist environment for the wound [14,15,16]. Foam dressings are normally made of hydrocellular or hydrophilic polyurethane. They can reduce healing time of wounds compared with traditional dressings [17,18]. Based on their high performance, the foam dressings were chosen to compare with superabsorbent spacer fabrics in this study.

The application of spacer fabrics as absorbent medical products has recently attracted some attention. Davies et al. [19] compared the absorbency and liquid spread of spacer fabrics knitted with different absorbent yarns. A comparison of warp-knitted spacer fabrics with commercial wound dressings indicated that the substitution of the absorbent layer of advanced wound dressing by spacer fabric was possible [20]. In our previous study, the potential of the application of spacer fabrics as a wound dressing was explored [21,22]. The study showed that spacer fabric-based dressings had much better air permeability than foam dressings. Their absorbency was higher than alginate dressings. Another study also showed that spacer fabrics could absorb exudate and kill bacteria within the dressing [23]. However, the absorbency of the designed spacer fabric was much lower than one of the foam dressings, and the water vapor permeability of the designed spacer fabric was higher than that of foam dressing. According to the requirements for exuding wound dressing, the low absorbency and high water vapor permeability would prolong the healing process. In this study, superabsorbent spacer fabrics were designed and fabricated by using superabsorbent yarns as spacer yarns to increase the absorbency and moisture retention of wound dressing and to improve the moist-healing process of exuding wound.

## 2. Materials and Methods 

### 2.1. Fabrication of Superabsorbent Spacer Fabrics

**Figure 1:**
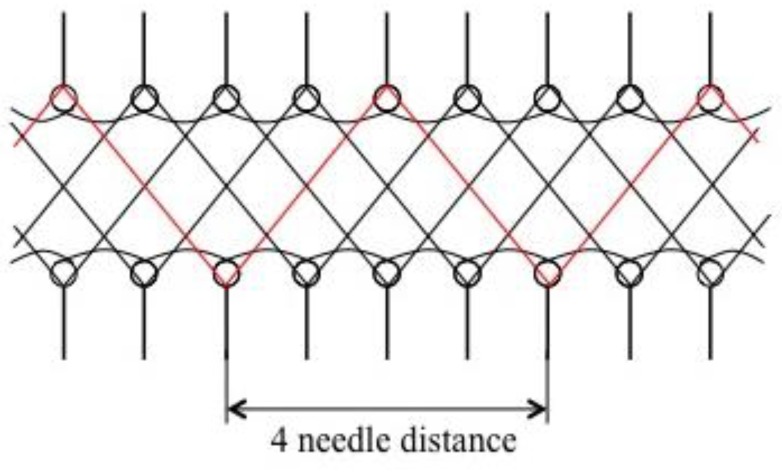
Spacer fabric structure with four-needle connecting distance.

A spacer fabric is composed of three layers, i.e., two outer layers and one spacer layer. As wound dressings should quickly absorb great amounts of exudates, superabsorbent yarns were selected to knit the spacer layer of the fabric. In order to immediately guide the fluid and moisture into the spacer layer, the hydrophobic yarn was used to knit the two outer layers. Due to the large size of the superabsorbent yarns, spacer fabrics were knitted on a 7-gauge STOLL CMS 530 (STOLL, Reutlingen, Germany) computerized flat knitting machine. The hydrophobic yarn used was a polyester/spandex (100D/40D) yarn provided by Tailin (Zibo, China) Textile Co., Ltd. As the surface yarns are elastic, the number of surface yarns would affect the thickness and density of the spacer fabrics. Therefore, one single and two polyester/spandex yarns were used, respectively. Since the content of superabsorbent fiber affects absorbency, two types of superabsorbent yarns with different thicknesses were used as spacer yarns. Both types of yarns were from Technical Absorbents Ltd., (Grimsby, UK) with code CA–1800HS (superabsorbent fiber, namely SAF) and CA-4500HS (superabsorbent fiber thicker, namely SAFT), respectively. The spacer layer (distance zone) was knitted with four connecting distances as shown in Figure 1.

**Figure 2:**
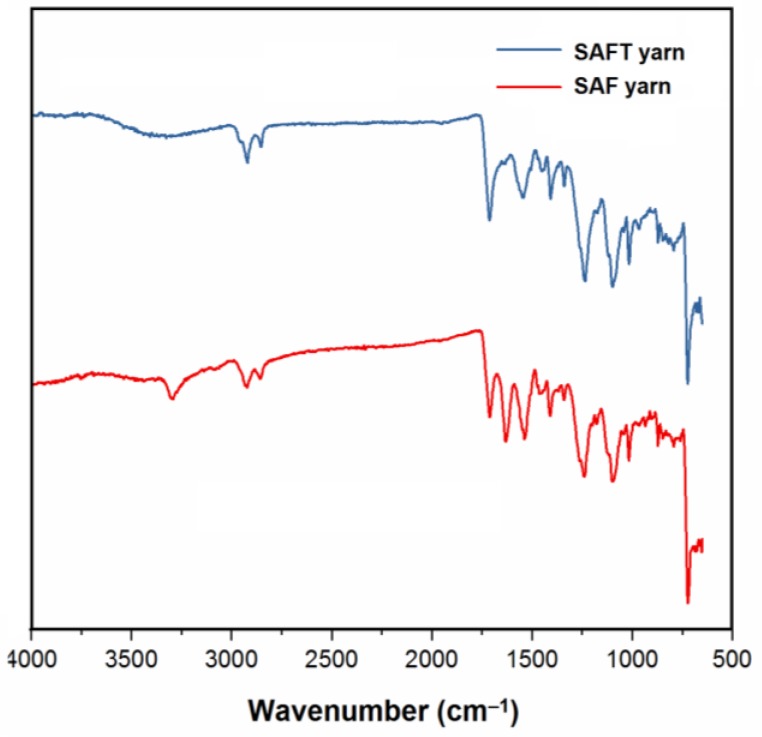
FT-IR spectra of SAF yarn and SAFT yarn.

The main properties of the superabsorbent yarns used are listed in Table 1. While the yarn diameter, composition, minimum breaking strength, and maximum breaking extension were provided by the supplier, the yarn linear density was measured according to standard ASTM D3818. The superabsorbent yarns were blended together with superabsorbent fiber SAF^TM^, PES fiber, and PA fiber. The SAF^TM^ was the copolymer of acrylic acid, sodium acrylate, and methyl acrylate [24], which was crosslinked into the polymer during the free-radical-initiated addition polymerization. The chemical structures and function groups of superabsorbent yarns were characterized by a Perkin-Elmer Spectrum 100 on a FT-IR spectrometer in a range of 650–4000 cm^–1^ at room temperature (Figure 2). The absorption band at 3252 cm^–1^ was the stretching vibrations of O–H in acrylic groups, which were the key for the absorbency of the yarns. The absorption band at 2921 cm^–1^ was due to the asymmetric and symmetric stretching vibrations of –CH_2_. The absorbing peak at 1708 cm^–1^ is due to the stretching vibration of C=O bond in acrylic groups. There was a peak at 1627 cm^–1^ attributed to C=C stretching. 

It can absorb up to 200 times of its own weight in demineralized water and circa, and 60 times its own weight in saline (0.9% *w*/*v* NaCl) at an extremely fast absorption rate [25]. SAF™ is white and odorless. It looks and handles like a textile fiber and can be easily converted into spun yarns. SAF^TM^ is non-irritant and FDA approved [25].

**Figure 3:**
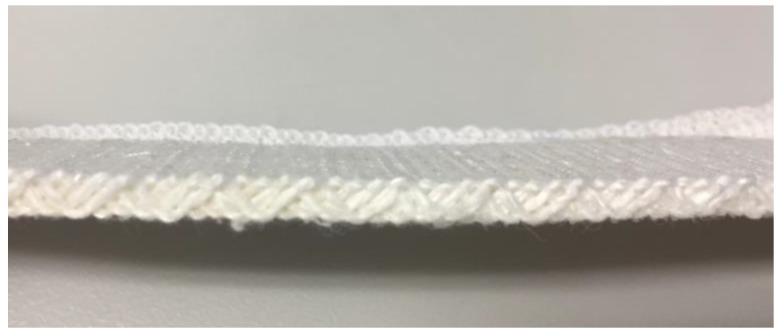
Photograph of a typical fabricated spacer fabric.

After knitting, a steaming treatment was carried out by using an HSL-611 steam iron produced by NAOMOTO Corporation in Japan. The treatment made the spacer fabrics shrink due to shrinkage of the surface elastic yarns. The treated fabric samples were then conditioned at 20 °C and 65% RH for a week to obtain fully-relaxed fabrics with stable dimensions before further use. The photograph of a typical fabricated superabsorbent spacer fabric is shown in Figure 3.

By combining two numbers of surface yarns and two kinds of superabsorbent spacer yarns, four types of fabrics were produced. The fabric codes and the specifications of the spacer fabrics, including areal mass, thickness, density, porosity, and surface stitch density, are listed in Table 2. Except thickness, the differences of all other properties between the four kinds of the spacer fabrics were significant (*p* < 0.05). In the fabric codes, letters and Arabic numerals are used to indicate the type of absorbent fiber for knitting the spacer (absorbent) layer and the number of yarn for knitting the surface layers, respectively. While the fabric thickness was measured by using PEACOCK model H thickness tester, the fabric density was calculated according to Equation (1).

Density (g/cm^3^) = *W*_m_/*V*_m_ (1)

where *W*_m_ is the mass of fabric (g), and *V*_m_ is the total volume of fabric (cm^3^).

As the superabsorbent yarns are much thicker and heavier than the surface yarns, only the superabsorbent yarns were taken into consideration when calculating porosity of the spacer fabric. The density of the blended superabsorbent yarns ρ was calculated according to Equation (2):

*ρ* (g/cm^3^) = Σ (*ρ*_i_ × *P*_i_ ) (2)

where *ρ*_i_ is the fiber density (g/cm^3^), *P*_i_ is the fiber percentage in the blended yarn.

The porosity of samples was determined according to Equation (3):

Porosity (%) = (*V*_m_ − *V*_p_)/*V*_m_ × 100 = (1 − (*W*_m_/ρ)/*V*_m_) × 100 (3)

where *V*_m_ is the total volume of fabric (cm^3^), *V*_p_ is the actual volume of fibers (cm^3^), *ρ* is the density of the blended superabsorbent yarns (g/cm^3^), and *W*_m_ is the mass of fabric (g).

### 2.2. Property Evaluation

**Figure 4:**
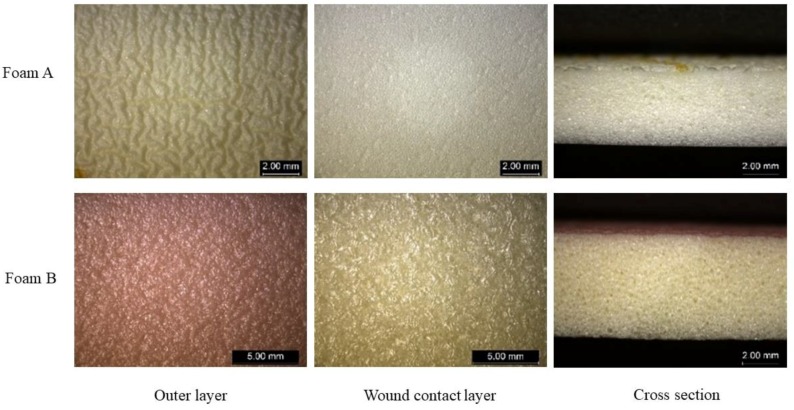
Photographs of the foam dressings from market.

This study evaluated the wound dressing properties of the superabsorbent spacer fabrics, including wettability, absorbency, air permeability, water vapor permeability, thermal insulation and conformability. In order to show their potential to be used as exuding wound dressings, the superabsorbent spacer fabrics were compared with commonly used dressings made of polyurethane foams. The details of those commercial dressings are listed in Table 3, and the photographs of their outer layer, wound contact layer and cross section are shown in Figure 4. Foam A is a type of soft and flexible foam dressing which has superior adsorption. It has a long wear time and minimized risk of maceration and leakage due to the superior absorption capacity [26]. Foam B can effectively manage fluid to create a moist wound healing environment [27]. Since the spacer fabrics are not adhesive, the chosen foam dressings are non-adhesive too. As shown in Table 3, the foam dressings are thinner and lighter than the spacer fabrics.

#### 2.2.1. Wettability Test 

Wetting time of samples was tested to evaluate their wettability according to standard AATCC 79. This method was used to measure the time spent by a fabric on absorbing liquid. The test was carried out in a standard atmosphere. During the test, one drop of distilled water was dripped from 10 ± 1 mm above the sample. The time required for the liquid to lose its specular reflectance under a spotlight was measured using a stopwatch. The drop was gradually absorbed, and the tiny mirror diminished. The watch was stopped at the instant when the reflectance entirely vanished. Five water drop sites were tested for each sample and the average values were reported.

#### 2.2.2. Absorbency Test 

The absorbency test aimed to measure the liquid retained in a dressing. The absorbency is a key factor for wound healing. It was tested following British Standard 7959. During the test, a sample of 10 cm × 10 cm (100 cm^2^) was placed on the surface of distilled water. The sample was gradually wetted and sank into the water. After reaching visual saturation, the sample was immersed into water for two minutes, and then removed and drained for 30 s. The absorbency of sample was finally calculated according to Equation (4). Five specimens were tested for each type of fabric.

Absorbency (g/100 cm^2^) = *W*_1_ − *W*_0_ (4)

where *W*_0_ is the weight of the dry sample (g/100 cm^2^); and *W*_1_ is the weight of the sample after absorption (g/100cm^2^).

#### 2.2.3. Air Permeability Test 

The air permeability was evaluated on a SDL M021S type air permeability tester according to standard ASTM D 737. A sample was placed on the circular testing head. Its wound contact layer was facing downward to firstly contact with the air. This test was carried out under a water pressure difference of 100 Pa. The result was recorded in SI units and then calculated as mL/s/cm^2^. The maximum value of the tester is 78.040 mL/s/cm^2^, and the minimum value is 0.020 mL/s/cm^2^ at 100 Pa. Five samples were evaluated for each dressing.

#### 2.2.4. Water Vapor Transmission Rate (WVTR) Test 

The WVTR test was conducted according to British Standard 7209. Samples were cut into a circular form with the same outer diameter of the testing dish used. The inner diameter of testing dish was 83 mm. 46 cm^3^ of water was put into the dish, and the prepared specimen was fixed onto the rim of the testing dish (wound contact layer facing down) by using adhesive cement. In the beginning, the distance from the wound contact layer of the sample to the surface of water was 10 ± 1 mm, and the mass of the dish, together with the water and sample, was measured. Then, the dish was placed on a turntable rotating with a speed of 2 r/min for 24 h to avoid the formation of still air layers above the dish. After 24 h the mass of the dish was measured again. The amount of water evaporated per m^2^ per 24 h was calculated according to Equation (5). Three repeating tests were conducted for each type of fabric.

WVTR (g/24h/m^2^) = (*M*_0_ − *M*_1_)/*A* *A* = π*d*^2^/4 (*d* = 0.083m) (5)

where *M*_0_ and *M*_1_ are the masses of the dish together with water and fabric sample at the beginning and after 24 h of test (g); *A* is the evaporation area (m^2^); and *d* is the inner diameter of dish (m).

#### 2.2.5. Thermal Insulation Test 

A KES-F7 Precise and Fast Thermal Property-Measuring Instrument Thermo Lab II was used to test the thermal insulation. A sample of 20 cm × 20 cm was placed onto the testing area of the instrument, and the q-max values (warm/cool feeling evaluation value) with and without samples were measured, respectively. The test for each type of sample was repeated for five times. The rate of heat keeping was calculated according to Equation (6):

*Q* (%) = (1 − *Q*_2_/*Q*_1_) × 100 (6)

where *Q*_1_ and *Q*_2_ are, respectively, the q-max values without and with sample placed on testing area (J/°C).

#### 2.2.6. Conformability Test 

The extensibility and permanent set conformability of the spacer fabrics and foam dressings were tested according to BS EN 13726-4—Test Method for Primary Wound Dressings—Conformability. The extensibility tests were carried out on an Instron 4411 tester. The weft direction of the spacer fabrics was measured. For the commercial dressings, there was no difference between directions. Specimen with 25 mm width and 80 mm clamping length (two parallel marks were made on the specimen along the edge of clamp, so the original distance between the two marks was 80 mm (*L*_1_)) was extended by 20% at an extension rate of 300 mm/min. The maximum load (ML) was recorded. The specimen was held at this extension for 60 seconds and then removed from the jaws. The distance between the two marks on the specimen (*L*_2_) was remeasured after a relaxation of 300 seconds. The extensibility was calculated using Equation (7):

Extensibility (N/cm) = ML/2.5(7)

where ML is the maximum load when the sample is stretched to 20% (N). 

The permanent set was calculated using Equation (8):

Permanent set (%) = (*L*_2_ − *L*_1_)/*L*_1_ × 100 (8)

where *L*_1_ is the distance between the two marks before elongation (mm); and *L*_2_ is the distance between the two marks after relaxation (mm).

#### 2.2.7. Statistical Analysis 

One-way analysis of variance (ANOVA) was performed using Matlab software. The *p* values were calculated to evaluate the difference between testing samples. The higher the *p* value is, the lower the significance is. In this study, *p* < 0.05 indicated that the difference was statistically significant.

## 3. Results and Discussion

### 3.1. Wettability

**Figure 5:**
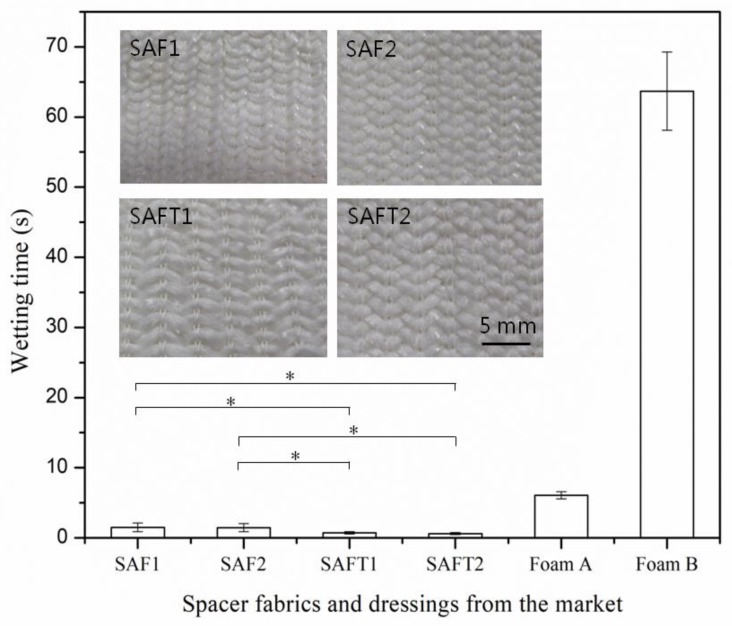
Wetting time of the spacer fabrics and foam dressings from the market. The photos of the surfaces of superabsorbent spacer fabrics are shown to visualize their difference. * *p* < 0.05.

The wetting time of the superabsorbent spacer fabrics and foam dressings was measured and the results are shown in Figure 5. Wetting time represents the wetting speed of dressing. A shorter wetting time indicates a better wettability. As can be seen from Figure 5, all the superabsorbent spacer fabrics had much faster wetting speeds (less than 2 s) than the foam dressings from the market (6.04 s for Foam A and 63.69 s for Foam B). The reason was that the loop heads of the superabsorbent spacer yarns formed absorbent dots on the fabric surface made of hydrophobic polyester/spandex yarn. Those dots directly contacted liquid, quickly guiding the liquid into the middle layer. Another reason was that, unlike polyurethane foams, the capillary action of textile fibers helped wetting, which in turn reduced the risk of skin maceration. For the foam dressings, Foam B required much longer time (more than 60 s) to be wetted than Foam A because its wound contact surface was covered with a non-adherent film (Figure 4) which weakened the wettability. For the superabsorbent spacer fabrics, their wetting time was less than two seconds, indicating that they could absorb liquid into its body within a very short time. The wetting speeds of the spacer fabrics made of the thicker SAFT yarns were faster than those of the spacer fabrics made of SAF yarns (*p* < 0.05) due to lower surface stitch densities of SAFT1 and SAFT2 fabrics (Table 2, Figure 5). 

Fast wetting speed is essential because prolonged exudate exposure can be harmful to the wound healing process [28]. Caustic elements contained in wound exudates significantly delay the proliferation of fibroblast cells, which inhibits the synthesis of extracellular matrix and collagen [29,30]. Therefore, long-term exposure to wound exudate containing caustic elements facilitates epidermal extracellular matrix and growth factor breakdown [31]. The advantage of a surperabsorbent spacer fabric in wettability aids to rapidly transport harmful exudates away from the wound bed. 

### 3.2. Absorbency

**Figure 6:**
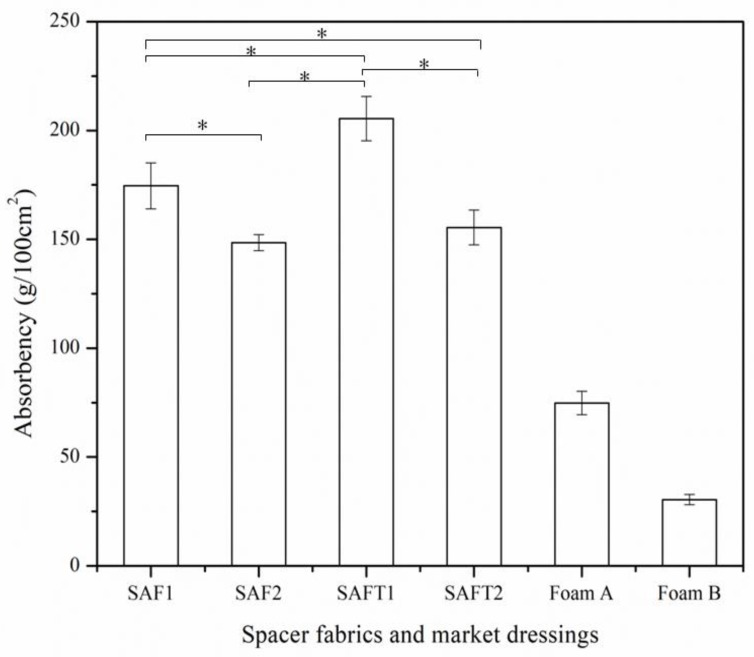
Absorbency of the spacer fabrics and foam dressings from the market. * *p* < 0.05.

The absorbency of the superabsorbent spacer fabrics and foam dressings is presented in Figure 6. It can be seen that the absorbency of the superabsorbent spacer fabrics was at least twice higher than that of the foam dressings. As mentioned before, the superabsorbent fiber made of superabsorbent polymer could absorb up to 200 times of its own weight in demineralized water and circa, and 60 times of its own weight in saline (0.9% *w*/*v* NaCl) at an extremely fast absorption rate [25]. Although the superabsorbent yarns consist of less than 30% superabsorbent fibers and more than 70% hydrophobic fibers (PES and PA fibers), their absorbency is much higher than hydrophilic and hydrocellular polyurethane foams. 

The high absorbency of the superabsorbent polymer contributed to the high absorbency of the spacer fabrics. The superabsorbent polymer was only slightly crosslinked so that the polymer chains could adopt widely spaced configurations to absorb large quantity of aqueous fluid. When superabsorbent fiber is in the presence of a liquid, hydrogen bond will be formed through the hydration of the acrylic acid groups. Meanwhile, the sodium is dissociated from the carbonyl group creating carboxyl (COO^−^) and sodium cation (Na^+^). Due to the same negative charge, the carboxyl groups begin to repel each other. As a result, the polymer chains uncoil and swell, allowing the polymer to interact with more water molecules. Some polymer chains are constrained due to the cross-linking. Water molecules can be drawn into the network across a diffusion gradient formed by the sodium neutralization of the carboxyl acid groups (–COOH) along the polymer backbone (–COO^−^Na^+^). Therefore, the water is tightly held in the network by hydrogen bonds.

The results also showed that the absorbency of the spacer fabrics made of the SAFT yarns was higher than that of the spacer fabrics made of the SAF yarns. As the SAFT yarns were much thicker than the SAF yarns, the spacer fabrics made of the SAFT yarns contained more superabsorbent fibers than the spacer fabric made of the SAF yarns, which increased the absorbency of the spacer fabrics made of the SAFT yarns. Because of their lower porosity, the spacer fabrics knitted with two surface yarns had lower absorbency than spacer fabrics knitted with single surface yarns (*p* < 0.05). 

**Figure 7:**
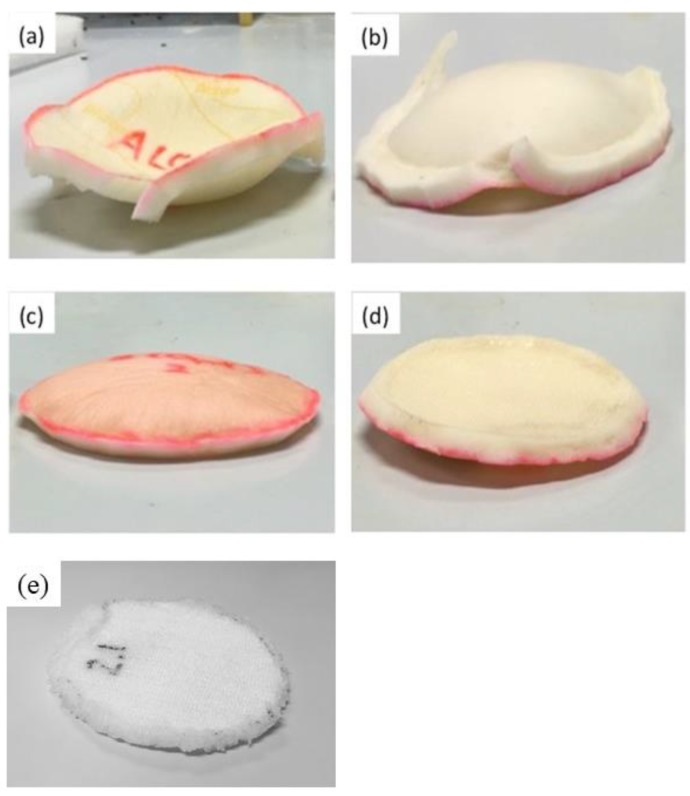
Shape changes of the foam dressings and spacer fabric after absorbing water: (**a**) the outer surface of Foam A; (**b**) the wound contact layer of Foam A; (**c**) the outer surface of Foam B; (**d**) the wound contact layer of Foam B, and (**e**) the superabsorbent spacer fabric.

Another issue that deserves attention was the deformation of the foam dressings after absorbing water. As shown in Figure 7, the foam dressings swelled after absorbing water as their thickness was increased. While Foam A was concaved towards the wound contact layer, Foam B was bulged toward the top layer. The main reason for the swelling was that the hydrophilic and hydrocellular polyurethane foams were combined with water and the stored water inside their body increased their volumes. When foams were fixed on the wound during the application, the swelled foams could not expand towards the edges, therefore, their shape was deformed. The deformed dressing would not fit well with the shape of wound surface, lowering the moisture of wound healing environment and causing dehydration and healing delay of wound. Differently, the spacer fabric after absorbing water was still in a flat form. The high porosity of spacer fabric gave enough spaces for swelled yarns. Therefore, the superabsorbent spacer fabric reduced the negative effects on the wound caused by the shape change. Although the superabsorbent yarns produce some gel after absorbing, SAF^TM^ is non-irritant and FDA approved. According to the toxicological information of SAF^TM^, mutagenic activity or irritation to skin or eye could not be found. No evidence of delayed contact hypersensitivity was observed and the acute oral toxicity was low (LD50 rat > 2000 mg/kg) [24]. Therefore, the superabsorbent yarns can be safely used in wound dressing.

As mentioned above, the caustic elements presented in wound exudates may inhibit the production of collagen and growth factors and therefore have negative impact on the wound healing process. The surperabsorbent spacer fabrics allow large amounts of fluid to be absorbed into the dressing, keeping adverse exudate away from the wound. The unmanaged wound exudate may also lead to peri-wound skin maceration, which slows down wound healing [32]. The superabsorbent spacer fabric could help to avoid peri-wound skin maceration. During the exuding period of wounds, the changing frequency of dressing mainly depends on the absorbency of exudates. Once a dressing reaches its maximum adsorption capacity, it should be changed to protect wound from harmful exudates and avoid maceration. Therefore, the dressing with higher absorbency could be changed fewer times. As the absorbency of the superabsorbent spacer fabric is much higher than foam dressings, the changing frequency of superabsorbent spacer fabric should be lower than that of foam dressings when applied on exuding wounds. In addition, the wound healing process is easily disturbed during the dressing changing, especially when the wound is adherent to the dressing. The reduction of the changing frequency lowers the risk of disturbance.

### 3.3. Air Permeability

**Figure 8:**
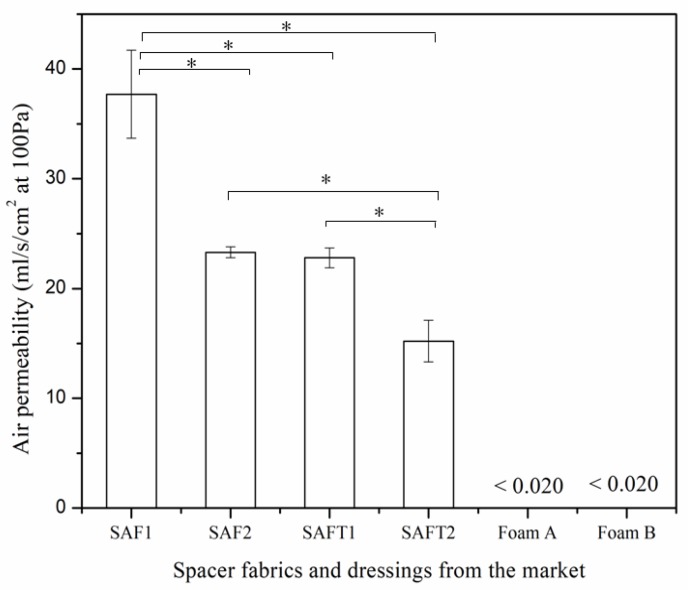
Air permeability of the spacer fabrics and foam dressings from the market. * *p* < 0.05.

**Figure 9:**
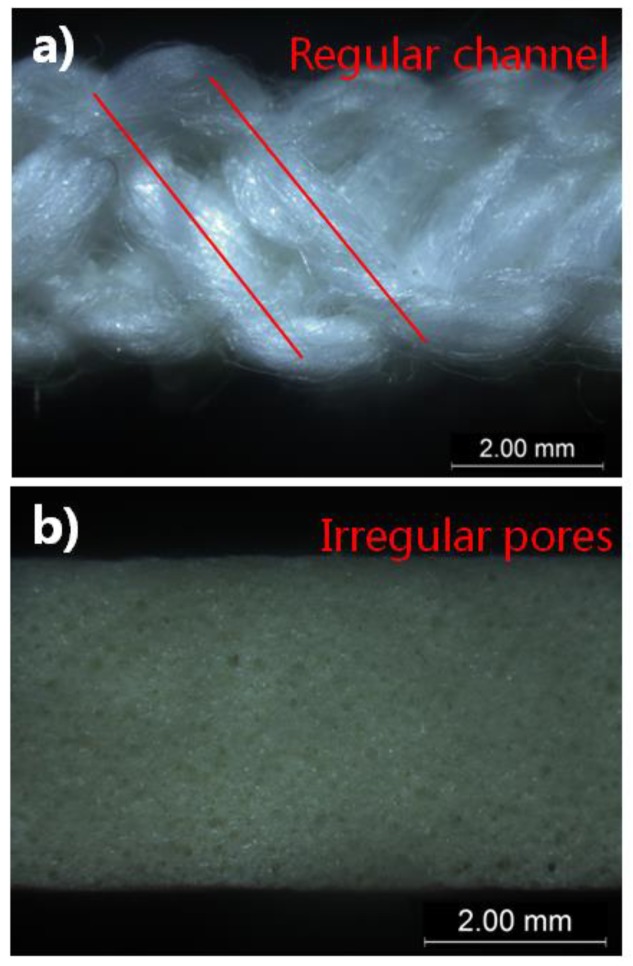
Light microscope pictures of cross-section of (**a**) superabsorbent spacer fabric, and (**b**) foam dressing.

The results of air permeability are shown in Figure 8. As the findings revealed, the air permeability values of the foam dressings were lower than 0.020 mL/s/cm^2^ at 100 Pa, exceeding the minimum value that the SDL M021S air permeability tester could measure. This indicated that the air permeability of the foam dressings was not comparable to that of the superabsorbent spacer fabrics. Although the superabsorbent spacer fabrics were thicker than the foam dressings, the foam dressings were much denser than the spacer fabrics according to their cross-section pictures, as shown in Figure 9. In addition, the pores were irregularly distributed in the polyurethane foams, whereas the spacer fabrics consisted of repeating units with regular channels. The regularly-arranged openings in the spacer fabric structure allowed air to flow freely and facilitated air circulation. Furthermore, the non-adherent film on the surface of Foam B reduced its gas permeability. 

The rate of wound healing may be reduced by the limited oxygen supply. This suggests that the wound healing may be promoted by increasing oxygen exchange [7,8]. A permeable dressing could help to increase collagen production when compared with non-porous dressing [33]. Air exchange also improves the fibroblast proliferation, re-epithelialization, and poly-morphonuclear cell functions [6,34]. In addition, as anaerobic bacteria were reported to cause the production of volatile odorous molecules [11,35], a wound dressing should permit diffusion of air to reduce wound malodor. The superabsorbent spacer fabrics with high air permeability could provide adequate wound oxygenation.

### 3.4. Water Vapor Permeability

**Figure 10:**
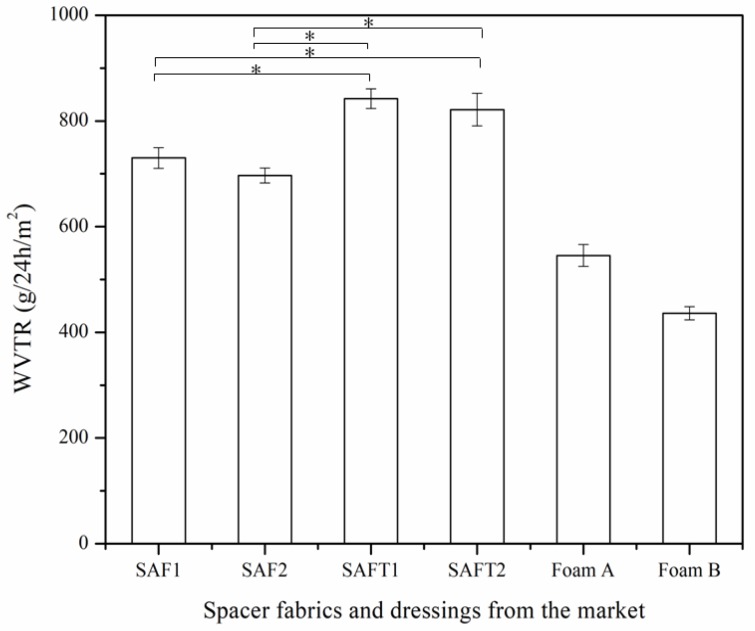
WVTRs of the spacer fabrics and foam dressings from the market. * *p* < 0.05.

The WVTRs of the spacer fabrics and foam dressings from the market are displayed in Figure 10. A higher WVTR indicates a faster evaporation from the tested sample. To maintain a moist environment for wound healing, a relatively low moisture evaporation is required for a wound dressing. The water vapor permeability depends not only on the density but also on the moisture absorbency of the material. A lower density leads to a higher WVTR, and a higher moisture absorbency brings a lower WVTR. The results showed that spacer fabrics knitted with SAFT spacer yarn had higher WVTRs than the fabrics knitted with SAF yarn (*p* < 0.05). Although the density of the superabsorbent spacer fabrics was much lower than that of the foam dressings, the difference of the WVTRs between the superabsorbent spacer fabrics and foam dressings was not so large. This is attributed to the high moisture absorbency of the superabsorbent yarns, which lower the evaporation of superabsorbent spacer fabrics.

Even though the superabsorbent spacer fabrics had higher evaporation than the foam dressings, the WVTR should not be considered the only indicator of efficacy for a wound dressing. Contrary to the requirement of high air permeability, wound dressing needs low WVTR. It is not easy to realize a higher air permeability and a lower WVTR at the same time because higher porosity would introduce higher air permeability, as well as higher WVTR. The high moisture absorbency of the superabsorbent spacer fabric helped to reduce the WVTR. A dressing is considered to be moisture retentive when its moisture vapor transmission rate is less than 840 g/24 h/m^2^ [36]. Therefore, the superabsorbent spacer fabrics SAF1 and SAF2 could be regarded as occlusive moisture retentive dressings as their WVTRs did not exceed this value.

A low WVTR may inhibit dry scab formation and facilitate re-epithelialization. However, the evaporation should not be too low to avoid infection and maceration. The WVTR of wound dressing was supposed to be higher than that of normal skin (200–500 g/24 h/m^2^) [37]. It was also reported that the spacer fabrics had better moisture retention than the foam dressings when the test was carried out after absorbing [23]. The superabsorbent spacer fabrics could maintain a moist environment to enhance wound healing.

### 3.5. Thermal Insulation

**Figure 11:**
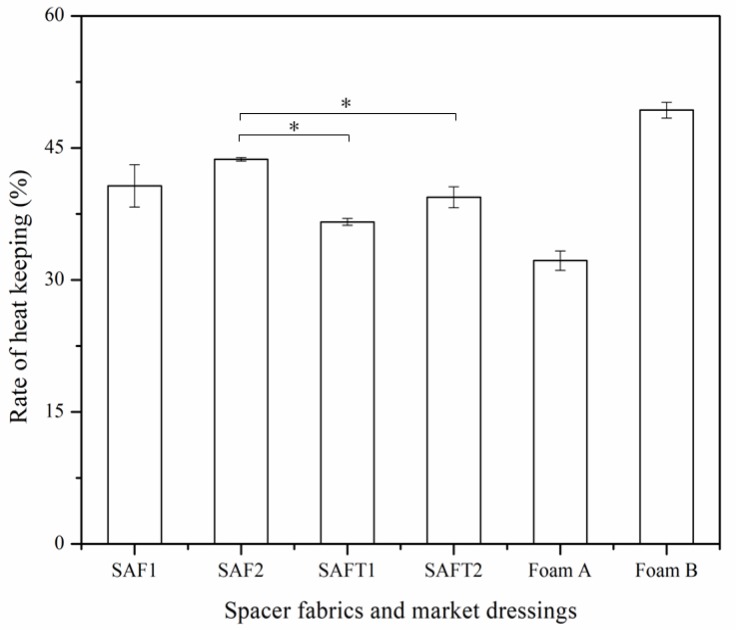
Rate of heat keeping of spacer fabrics and foam dressings from the market. * *p* < 0.05 .

The thermal insulating property was evaluated by measuring the rate of heat keeping (Figure 11). A higher heat keeping rate reflects a better heat retention property. The amount of heat loss of the spacer fabrics and foam dressings from the market were tested on a heat plate when a constant wind was continuously applied to the wound contact surface. As the thermal conductivity of air is low, the ability to keep still air inside the material mainly decides its heat retention. The results showed that the heat insulation of the superabsorbent spacer fabrics was higher than Foam A but lower than Foam B. 

Heat loss occurs when a wound covers a large area, which requires an insulating dressing to keep the wound in a temperature similar to that of the normal human body. The warm healing environment helps to optimize cellular function. Hypothermia may cause a relative vasoconstriction, slowing down the oxygen delivery to phagocytes and therefore reduces the mitotic activity and growth factor production [3]. Foam dressings could provide thermal insulation to the wound [38]. The heat keeping rate of the superabsorbent spacer fabrics was around 40%, which was located between the two kinds of the commercial foam dressings. This indicated that the superabsorbent spacer fabric had suitable thermal insulation for wounds. 

### 3.6. Conformability

**Figure 12:**
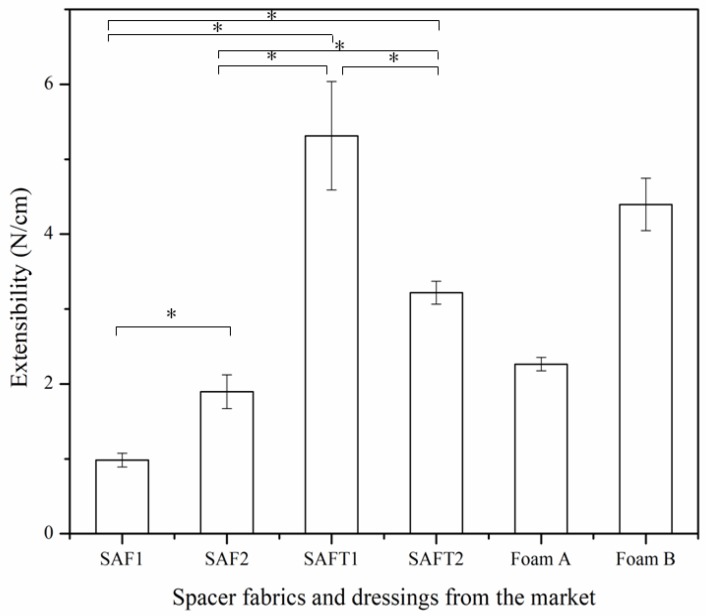
Extensibility of the spacer fabrics and foam dressings from the market. * *p* < 0.05. .

**Figure 13:**
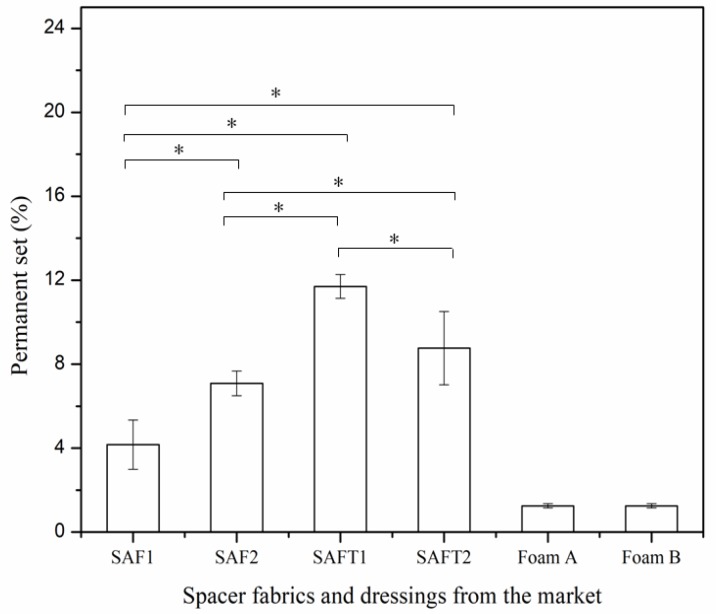
Permanent set of spacer fabrics and foam dressings from the market. * *p* < 0.05. .

The conformability of the spacer fabrics and foam dressings was evaluated by the extensibility (Figure 12) and permanent set (Figure 13). A high value of extensibility means that a high force is required to deform a material for a given extension. The higher the value is, the poor the extensibility is. It can be seen that the extensibility of SAF1 and SAF2 was better than that of the foam dressings, as they required less force to be deformed, making them more suitable for using in movement regions of the body. The permanent set represents the increase in the length of a sample after stretching and relaxing. The results of permanent set indicated that the foam dressings had better elastic recovery than the superabsorbent spacer fabrics.

As wound dressings contact with human skins during use, they should be comfortable and conformable. When a dressing is applied to a region of movement of the human body, for example over a joint, it should provide sufficient freedom to the joint to move. A dressing which is easily extended and can return closely to its original length after extension would be more comfortable for the patient to wear. Spacer fabrics SAF1 and SAF2 were easily stretched with low force, but their elastic recovery was lower than the foam dressings. As a result, the conformability of the superabsorbent spacer fabrics was comparable to the foam dressings from the market.

## 4. Conclusions

Four types of superabsorbent spacer fabrics were fabricated for being used as exuding wound dressings and their wettability, absorbency, air permeability, water vapor permeability, thermal insulating property, and conformability were tested and compared with those of the polyurethane foam dressings from the market. From the testing results, it can be concluded that the superabsorbent spacer fabrics had faster wetting speed, better absorbency, higher air permeability, and comparable water vapor permeability, heat insulation, and conformability. The study suggests that the superabsorbent spacer fabrics could be suitable candidates for exuding wound dressings to improve the moist healing environment and facilitate the wound healing process. 

## Figures and Tables

**Table 1:** Properties of superabsorbent yarns.

Yarn designation		SAF	SAFT
Absorbency (g/g)		35	32
Linear density (dtex)		2477	5071
Diameter (mm)		0.85	1.45
Minimum breaking strength (N)		18	100
Maximum breaking extension (%)		15	15
Composition (%)	Superabsorbent fiber	26.2	27.8
	PES fiber	61.5	65.4
	PA fiber	12.3	6.8

**Table 2:** Details of produced spacer fabrics.

Fabric Code		SAF1	SAF2	SAFT1	SAFT2
Number of surface yarns		1	2	1	2
Type of spacer yarn		SAF	SAF	SAFT	SAFT
Fabric thickness (mm)		6.271 (± 0.288)	6.227 (± 0.187)	6.423 (± 0.127)	6.048 (± 0.257)
Areal mass of fabric (g/cm^2^)		0.159 (± 0.004)	0.163 (± 0.014)	0.211 (± 0.009)	0.227 (± 0.004)
Density (g/cm^3^)		0.253 (± 0.006)	0.261 (± 0.022)	0.328 (± 0.013)	0.376 (± 0.006)
Porosity (%)		79.7 (± 0.2)	79.0 (± 0.5)	73.8 (± 0.8)	70.0 (± 0.4)
Surface stitch density	Course direction (wales/cm)	3.62 (± 0.08)	3.96 (± 0.15)	2.78 (± 0.08)	2.94 (± 0.05)
	Wale direction (courses/cm)	7.62 (± 0.30)	6.76 (± 0.22)	5.86 (± 0.15)	6.02 (± 0.04)
	Stitch density (loops/cm^2^)	27.58 (± 1.16)	26.75 (± 0.78)	16.29 (± 0.55)	17.7 (± 0.42)

Note: Standard deviations are given in parentheses.

**Table 3:** Details of the wound dressings from the market.

Code	Base material	Product name & brand	Areal mass (g/cm^2^)	Thickness (mm)
Foam A	Hydrophilic polyurethane foam	Biatain non-adhesive foam dressing from Coloplast	0.0754 (± 0.0023)	4.65 (± 0.06)
Foam B	Hydrocellular polyurethane foam	Allevyn non-adhesive foam dressing from Smith & Nephew	0.0680 (± 0.0014)	5.70 (± 0.05)

Note: Standard deviations are given in parentheses.
